# Diabetic *pdx1*-mutant zebrafish show conserved responses to nutrient overload and anti-glycemic treatment

**DOI:** 10.1038/srep14241

**Published:** 2015-09-18

**Authors:** Robin A. Kimmel, Stefan Dobler, Nicole Schmitner, Tanja Walsen, Julia Freudenblum, Dirk Meyer

**Affiliations:** 1 Institute of Molecular Biology/CMBI; Leopold-Francis University of Innsbruck, Technikerstrasse 25, A-6020 Innsbruck, Austria

## Abstract

Diabetes mellitus is characterized by disrupted glucose homeostasis due to loss or dysfunction of insulin-producing beta cells. In this work, we characterize pancreatic islet development and function in zebrafish mutant for *pdx1*, a gene which in humans is linked to genetic forms of diabetes and is associated with increased susceptibility to Type 2 diabetes. *Pdx1* mutant zebrafish have the key diabetic features of reduced beta cells, decreased insulin and elevated glucose. The hyperglycemia responds to pharmacologic anti-diabetic treatment and, as often seen in mammalian diabetes models, beta cells of *pdx1* mutants show sensitivity to nutrient overload. This unique genetic model of diabetes provides a new tool for elucidating the mechanisms behind hyperglycemic pathologies and will allow the testing of novel therapeutic interventions in a model organism that is amenable to high-throughput approaches.

Diabetes mellitus, characterized by impaired function of pancreatic beta cells, is a major health problem affecting an ever-increasing proportion of the world’s population^1^. In Type 1 diabetes, there is immune-mediated destruction of beta cells, leading to full dependence on exogenous insulin. Type 2 diabetes mellitus (T2DM) is the most prevalent form, accounting for more than 90% of all cases of diabetes^2^. It has a complex, multifactorial etiology, and features impaired beta cell function combined with peripheral insulin resistance^1^. Additional rare diabetes subtypes are monogenic and result from mutations in beta cell factors with important roles in both pancreatic development and mature function^3^. Dominant genetic forms that become symptomatic during adolescence or early adulthood are also referred to as Maturity-Onset Diabetes of the Young (MODY), while neonatal diabetes generally manifests in the first 6 months of life, and can result from dominant as well as recessive mutations^2^,^4^. Current diabetes therapies include insulin supplementation and drugs that enhance insulin secretion or tissue responsiveness. Treatments provide some protection and amelioration of acute effects, but many have unwanted side effects, and they generally do not sufficiently prevent accumulating damage to major organ systems, which has serious medical consequences.

Animal models of diabetes are an indispensible tool for dissecting biological mechanisms and testing potential new therapeutic avenues. Zebrafish as a model organism offers the advantages of ease of maintenance, amenability to genetic and pharmalogical manipulation, and transparency, which enables *in vivo* imaging of disease processes. For many medical problems including diabetes, there is an urgent need for cost-efficient methods for discovering new drugs. Recent studies have highlighted similarities in organ physiology and metabolism between zebrafish and mammals^5^,^6^, highlighting the potential for developing new models of disease.

Previously described zebrafish embryos depleted for pancreas-related factors (using antisense morpholinos) include examples of beta cell deficiency^5^,^7^, but these approaches are limited by the transient and incomplete effectiveness of morpholino knock-downs or early-lethal developmental effects on other organ systems. Alternative methods for generating hyperglycemic zebrafish have relied on incubation in high-glucose solutions^8^,^9^,^10^, and surgical or toxin-mediated ablation^11^,^12^,^13^,^14^,^15^. With such interventions there is often variability in the responses of the animals, and the regenerative response of pancreatic beta cells following ablation precludes long-term studies^12^,^13^,^14^,^15^.

Pancreatic and duodenal homeobox 1 (Pdx1, also known as insulin promoter factor 1, IPF1) is of key importance for both pancreas development and mature beta cell function and survival^16^. In human cases of *PDX1* mutations, the degree of endocrine dysfunction, and the nature of hyperglycemic disease, depends on the precise genetic lesion^17^. Absence of PDX1 is the genetic lesion in the type 4 form of MODY (MODY4) in the heterozygous state and causes pancreatic agenesis in the homozygous state, while partial loss-of-function mutations increase the risk of developing Type 2 diabetes but do not directly cause the disease^1^,^18^,^19^,^20^,^21^. Recently, homozygous hypomorphic *PDX1* mutations have been uncovered in cases of neonatal diabetes, which variably features subclinical exocrine pancreas dysfunction^22^,^23^. Similar phenotypic gradations are recapitulated in mouse models featuring various combinations of *Pdx1* mutations^17^.

In zebrafish as in mouse, *pdx1* is expressed in pancreatic progenitors during development and expression is maintained in mature beta cells. Recent studies confirmed similar requirements for Pdx1 in beta cell development between zebrafish and mammals, specifically that Pdx1 is essential for formation of the later population (“second wave”) of definitive endocrine cells^24^. Zebrafish Pdx1 regulates embryonic glucose metabolism^10^,^25^, and activates insulin gene expression in reporter assays^26^.

In this work, we describe a *pdx1* mutant zebrafish recently generated through the Zebrafish Mutation Project^27^ as a new model of diabetes. These mutants, which have a null mutation in *pdx1*, can survive to adulthood as homozygotes, but have reduced body size and decreased viability. Analysis of the pancreas phenotype revealed that beta cells and insulin levels are markedly reduced and the exocrine pancreas is specified, but acinar differentiation is perturbed. Importantly, the persistently elevated glucose in *pdx1* mutants responds to antidiabetic drug treatment. Finally, using a high fat diet feeding protocol, we show that beta cells of *pdx1* mutants are nutrient sensitive and undergo increased apoptosis. Overall, this new vertebrate genetic model of diabetes will allow the testing of new therapeutic interventions and provides a novel tool to elucidate biological mechanisms behind the toxic effects of sustained high glucose.

## Results

### *Pdx1^sa280^* mutant zebrafish are viable but show decreased size

**Figure 1:**
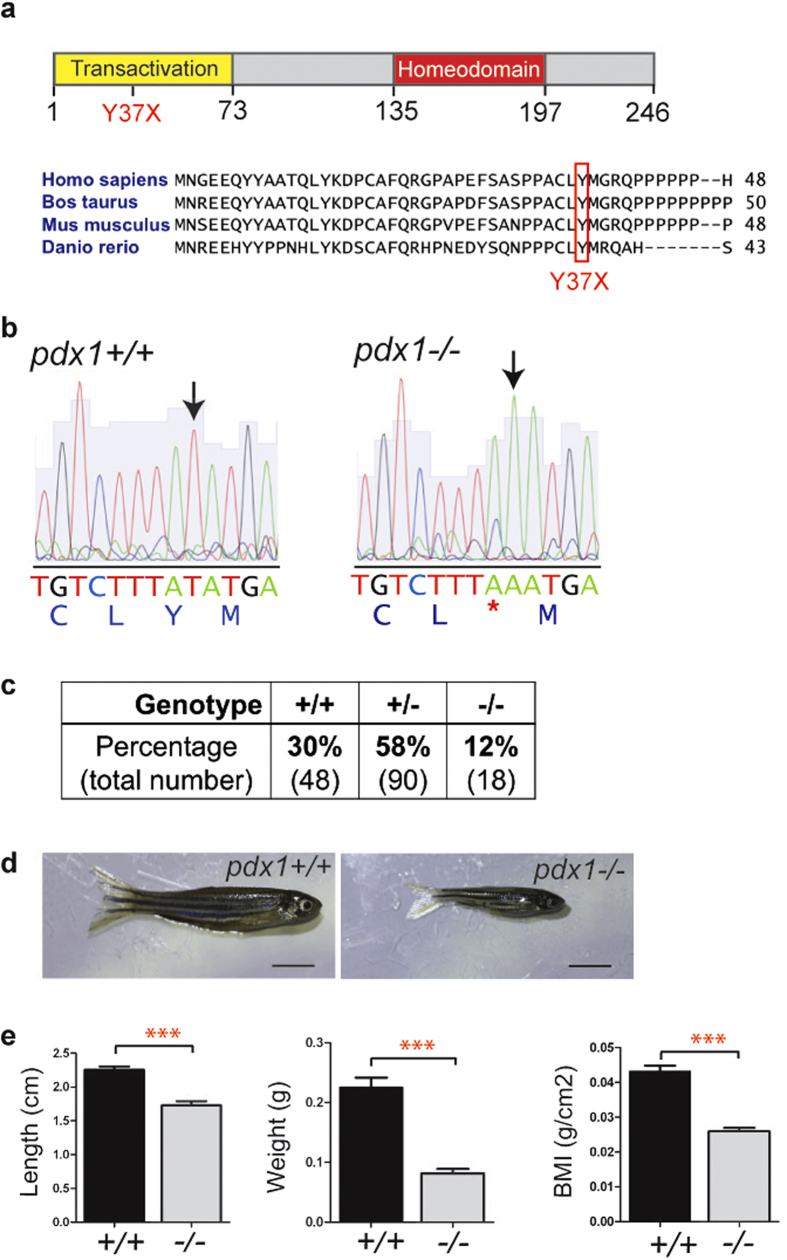
*Pdx1^sa280^* encodes the nonsense mutation Y37X. (**a**) Schematic of the zebrafish Pdx1 protein and sequence alignment showing the location of amino acid change Y37X resulting from mutant allele *pdx1*^*sa280*^. The mutated tyrosine at position 37, located within the highly conserved N-terminal transactivation domain of *pdx1,* causes a premature stop codon. (**b**) Sequencing of genomic DNA from 6-month-old adult offspring of a heterozygous incross revealed surviving homozygotes. (**c**) Genotype frequency at 6 months of incrossed heterozygotes. Surviving homozygous mutant *pdx1*^*−/−*^ fish were present at a lower than expected frequency. (Combined data from 4 independent crosses) (**d)** At 3 months of age, adult mutants (right) had normal morphology but reduced size compared to wild type controls (left). Scale bars, 0.5cm. (**e**) Homozygous mutants had decreased length, weight and BMI as compared to wild types. n(*pdx1*^*+/+*^) = 18, n(*pdx1*^*−/−*)^ = 19, ***p < 0.0001 (t-test).

Given the central role of Pdx1 in beta cell development and function, and the many advantages of zebrafish for modeling human disease, we characterized zebrafish with a mutation in *pdx1*^27^. The *pdx1* mutant allele designated *pdx1*^*sa280*^ yields a transcript with a premature stop at codon 37 (Y37X), which lies within the highly conserved N-terminal transactivation domain (Fig. 1a). Genotyping of adult offspring (6 months old) from an incross of *pdx1*^*sa280*^ heterozygotes revealed surviving homozygotes with the expected nucleotide alteration (Fig. 1b), however the homozygous genotype was present in reduced numbers as compared to the expected Mendelian ratios (Fig. 1c).

We further noted that homozygotes had decreased body size as compared to heterozygous and wild type siblings. At three months of age, body length, weight and body mass index (mass/length^2^) were all significantly reduced in homozygotes relative to heterozygous and wild types. This was observed for sibling fish raised in mixed-genotype tanks (Supplementary Fig. 1a–c), and was also the case for offspring from homozygous and sibling wild type incrosses raised in parallel at equal density (Fig. 1d,e). Therefore, the size difference was not due to a decreased ability of mutants to compete for food. Insulin deficiency is associated with decreased fetal growth, a feature commonly seen in neonatal diabetes^4^. However, analysis of free-swimming larvae at 12 days post-fertilization(dpf) showed normal morphology and no significant difference in body length (standard length, SL) between wild type and mutants (4.7 ± 0.3 mm for WT and 4.5 ± 0.3 mm for MU, p = 0.17, Supplementary Fig. 2a, b). Larvae then enter a period (∼one month) of high nutritional demand and rapid growth^14^,^28^. During this time we detected a size difference between mutants and wild types. At 5 weeks, body length was highly variable, but was decreased on average in mutant fish (SL11.2 ± 1.6 mm, n = 14) as compared to wild type (SL12.8 ± 3.6 mm, n = 12, p < 0.05, Mann-Whitney test; Supplementary Fig. 2c).

In mouse, the *Pdx1* knockout is characterized by failure of pancreas development^29^,^30^,^31^. To determine whether this is the case for the zebrafish *pdx1* mutant, we performed immunohistochemistry for pancreatic markers at 12dpf. We found that the exocrine pancreas developed in mutants, as indicated by immunostaining for CarboxypeptidaseA (CPA), as did an endocrine islet, as delineated by insulin (Ins)-positive cells in the head of the pancreas (Supplementary Fig. 2d).

**Figure 2:**
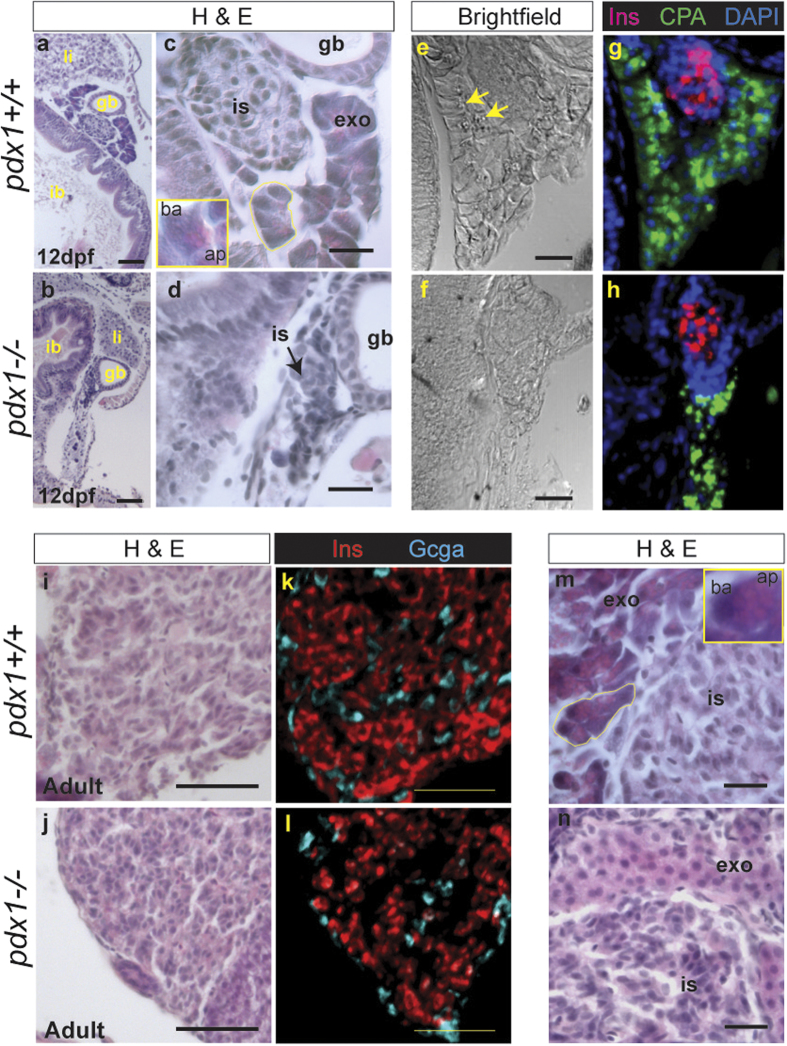
Pancreatic histology of *pdx1* mutants. (**a–d**) Gut region morphology in longitudinal-oblique sections of 12 dpf wild type (**a**,**c**), and *pdx1* mutant (**b**,**d**) larvae stained with hemotoxylin and eosin (H&E). (**a–d**) Pancreatic tissue is located between the intestinal bulb (ib) and gall bladder (gb). (li, liver) (**c**) In wild type embryos, exocrine cells (exo) adjacent to the islet (is) show clear polarity and acinar organization (yellow outline). Inset, close-up of single acinar cell, showing basophilic basal region (ba) and eosinophilic apical region (ap), where secretory granules are located. (**d**) In *pdx1* mutants cell polarity and acini are not apparent. In brightfield images of an adjacent region, exocrine secretory granules can be discerned in the wild type pancreas (**e,** arrows) in the region immunostained for CPA (green) (**g**). (**f**,**h**) CPA-positive exocrine tissue in the *pdx1* mutant appears disorganised. Beta cells of the islet are labeled by immunostaining for Ins (red). Nuclei are counterstained with DAPI (blue). ((**a**,**b**), scale bar = 50μm; (**c**–**f**), scale bar = 20μm) In adults, Ins + cells (red) occupy the core of the islet, while Gcga+ cells (cyan) are found mostly at the periphery in both controls and *pdx1* mutants (**i**–**l**). Scale bar=50μm. (**m**) Polarized exocrine cells (exo) arranged in acini (yellow outline) are found adjacent to the islet in controls Inset, close-up of a single acinar cell, showing basally (ba) located nucleus and eosinophilic apical region (ap), containing secretory granules. (**n**) In *pdx1* mutants, exocrine cells appear unpolarized and lack acinar organization. ((m,n) scale bar = 20μm)

To assess the exocrine pancreas in more detail, we performed histological analysis and immunostaining of paraffin sections of 12dpf larval pancreas. As in mammals, pancreatic acinar cells in zebrafish are characterised by a basally located nucleus and an eosinophilic apical region where secretory granules containing digestive enzymes are concentrated^32^ (Fig. 2c, inset, 2m, inset). Acinar cells and their lobular organization were discerned in wild type larvae in H&E stained sections (Fig. 2c). In immunostained sections, there was basal alignment of nuclei and the CPA signal was concentrated apically, colocalizing with exocrine granules (Fig. 2e,g). In *pdx1−/−* mutants, these histological features of exocrine tissue and acinar organization were not apparent (Fig. 2d), and the CPA immunostaining pattern was patchy and discontinuous (Fig. 2f,h). In adults (8–18 months old), the islet consisted predominantly of insulin (Ins)-expressing beta cells with peripherally located glucagon(Gcga)-expressing alpha cells (Fig. 2i–l). Polarized exocrine cells with acinar organization could be identified in H&E stained sections of wild type adult pancreas (Fig. 2m). In *pdx1−/−* mutant adults, exocrine cells lacked polarization and acinar organization was difficult to discern (n = 7/7, Fig. 2n).

### *Pdx1* mutation impairs pancreatic islet development

To evaluate the endocrine islet phenotype of *pdx1* mutant fish, pancreas development and function during embryonic and larval stages were examined. In zebrafish, early ‘first wave’ cells form the principal islet around 24 hpf through coalescence of dispersed precursors, while ‘second wave’ cells first appear around 3 dpf and account for the majority of beta cells in the adult^33^,^34^,^35^. Islet hormone-expressing cells could be detected in *pdx1* mutants at 36 hpf (Supplementary Fig.3), although cell number was reduced in mutants as compared to controls (46 ± 6 cells/embryo in controls compared to 40 ± 5 cells/embryo in mutants, p < 0.05, t-test).

**Figure 3:**
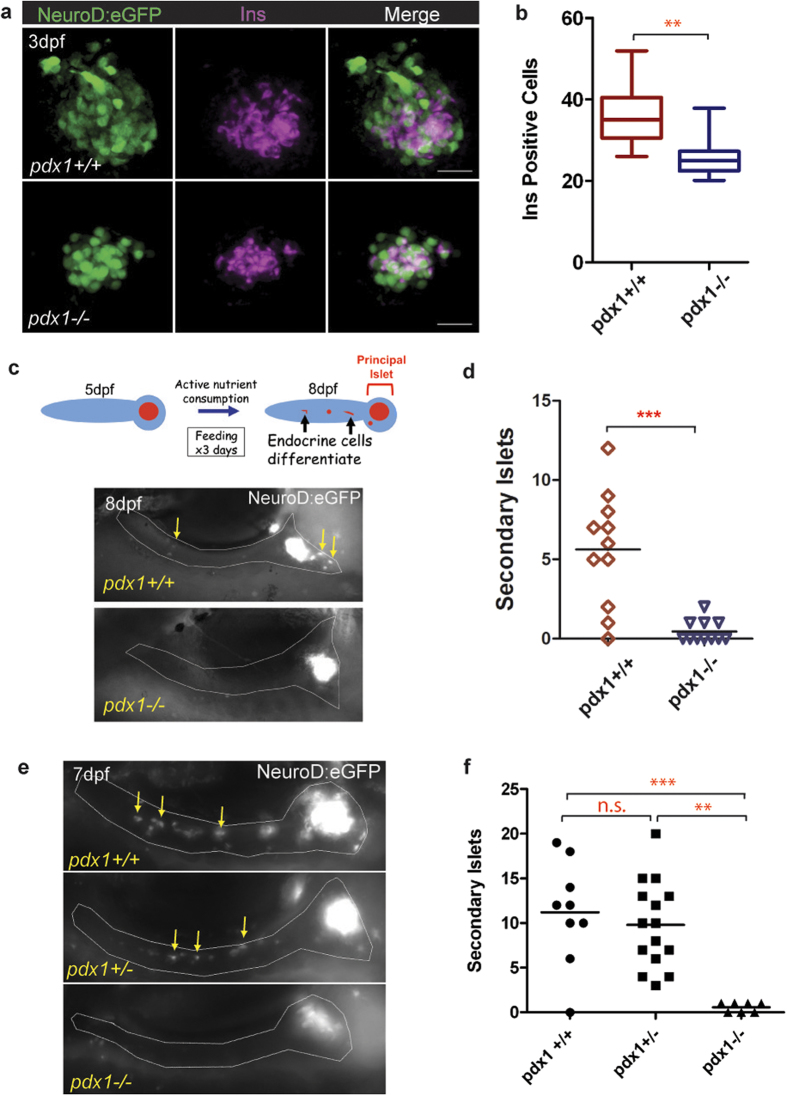
Islet formation but impaired expansion in *pdx1* mutants. (**a**) Immunostaining for insulin (Ins, magenta) and GFP protein (green) in wild type and mutant *NeuroD:eGFP+* embryos at 3 dpf. Maximum intensity projection (MIP) of confocal stacks. Scale bar, 20μm. (**b**) Number of Ins-expressing cells from embryos as shown in (**a**). (wt, n = 9; mu, n = 10) **p < 0.001 (t-test). The line indicates the median, boxes span the 25^th^ to 75^th^ percentile, whiskers the 10^th^ to 90^th^ percentile. (dpf, days post fertilization) (**c**, top) Schematic of experiment to evaluate feeding induction of secondary islets. Embryos were provided with nutrient powder (SDS100) from day 5 until day 7, and imaged live on day 8 to detect secondary islet cells. (**c**, bottom) NeuroD:eGFP+ endocrine cells are detected outside of the islet in control *pdx1*^*+/+*^ larvae (left), which are absent in *pdx1*^*−/−*^ mutants (right). The pancreas is outlined (gray) based on a simultaneously acquired brightfield image. (**d**) Quantitation of secondary islets formed in wild type (n = 11) and mutant (n = 11) larvae treated as in (**c**), ***p < 0.0001, t-test. (**e**) Representative 7 dpf *NeuroD:eGFP+* larvae treated with Ly411575 starting on day 4 and examined live by fluorescence microscopy. Heterozygous and control larvae, but not mutants, show robust induction of new GFP+ endocrine cells in the pancreatic tail (arrows). (**f**) Quantitation of second wave islet cells from *NeuroD:eGFP+* larvae treated as in (**e**). ***p < 0.0001; **p < 0.001; n.s., not significant (one-way ANOVA, Tukey Multiple Comparison test). n(*pdx1+/+*) = 8, n(*pdx1+/−*) = 13, n(*pdx1−/−*) = 7. In (c) and (e), the fluorescent signal in the prinicipal islet has been overexposed to reveal single cells and clusters in the surrounding pancreas. Single cells and clusters were quantitated as secondary islets.

To facilitate further studies, we generated compound transgenics which contained the *pdx1*^*sa280*^ mutant allele in combination with the *TgBAC(NeuroD:eGFP)nl1* transgene^36^ (hereafter referred to as *NeuroD:eGFP*) which is expressed in endocrine progenitors and differentiating endocrine cells^7^,^24^. Immunohistochemistry of 3.5 dpf embryos with an antibody specific to the N-terminal portion of the Pdx1 protein, in combination with an antibody to GFP, localized the islet and demonstrated the absence of detectable Pdx1 protein in the mutants (Supplementary Fig. 4). To further evaluate endocrine cell formation in *pdx1* mutants, we performed immunofluorescence staining of *NeuroD:eGFP*;*pdx1^−/−^* mutant and *NeuroD:eGFP*;*pdx1^+/+^* control embryos at 3 dpf. The overall size of the islet was markedly reduced in mutants, as was insulin expression (Fig. 3a). Insulin-producing cells were reduced 28%, from 36 ± 9 cells/embryo in controls as compared to 26 ± 5 cells/embryo in mutants (Fig. 3b).

As Pdx1 plays a role in islet cell fate allocation^37^,^38^, and loss of Pdx1 has been associated with cell identify transformations^39^,^40^, we assessed *pdx1* mutants for Glucagon(Gcga)-producing alpha cells at 72 hpf (Supplementary Fig. 5a–d). Alpha cell number was reduced from 29 ± 4 in controls to 23 ± 6 in mutants (p < 0.05, t-test, Supplementary Fig. 5e). While the cell numbers were reduced in mutants, the ratio of alpha to beta cells was not significantly changed (0.81 in control versus 0.93 in mutants, p = 0.18, t-test; Supplementary Fig. 5f).

The above data suggested that early endocrine cell specification was mostly intact in *pdx1* mutants, as previously reported in the *pdx1* knockdown^24^,^41^. However, the majority of beta cells arise after the onset of feeding at 5 dpf^14^, thus after developmental stages accessible to morpholino injection approaches. We next asked whether late-forming endocrine cells can differentiate in *pdx1* mutants. To look at new cell formation in response to nutrient stimuli, control and mutant embryos transgenic for *NeuroD:eGFP* were fed larval powder (SDS100 “fry food”) from 5 dpf until 7 dpf, and examined at 8 dpf. We quantitated as secondary islets eGFP-positive cells and clusters located outside the principal islet and in the pancreatic tail (Fig. 3c). Mutants showed minimal to no induction of new islet cells (0.5 ± 0.7), as compared to 5.6 ± 3.6 secondary islets in controls (Fig. 3d).

Second wave beta cells arise from pancreatic duct-associated progenitors that are maintained in an undifferentiated state by Notch signaling^34^,^35^. To test whether differentiation of these progenitors could be activated directly, we applied the Notch inhibitor Ly411575 to wild type, heterozygous, and mutant embryos transgenic for *NeuroD:eGFP*, to induce and visualize newly differentiating endocrine cells, as previously described^24^,^42^. We treated embryos from 4 dpf until 6 dpf, and examined embryos by live micropscopy for newly formed eGFP-positive cells and clusters, as described above. Virtually no new islet cells formed in mutants (0.6 ± 0.5 cells/embryo), compared to an average of 9.4 ± 5.2 secondary islets in heterozygotes and 11.4 ± 6.2 in wild types (Fig. 3e,f). *Pdx1* heterozygosity conferred no difference in islet cell forming potential as compared to the wild type state (Fig. 3e,f). It should be noted that far more cells can be induced to differentiate through pharmacological inhibition of Notch, as compared to differentiation under normal feeding conditions (Fig. 3f versus d).

The lack of Notch-induced endocrine cell differentiation could be due to absence of Notch-responsive duct cells, or due to an inability of these cells to differentiate into endocrine cells. To distinguish these possibilities, we analyzed the pancreatic duct by immunohistochemistry. We found that *pdx1* mutants formed pancreatic duct as defined by 2F11 antibody staining^42^ (Supplementary Fig. 6a, b). We further analyzed the cells of the duct by examining Nkx6.1 expression, which has been shown to colocalize with pancreatic Notch-responsive cells^43^. In 6 dpf larvae, Nkx6.1-expressing cells were found surrounding the islet and throughout the length of the pancreatic tail (Supplementary Fig. 6c, c’, e). Nxk6.1-positive cells were similarly distributed within the pancreatic head and tail of *pdx1* mutants (Supplementary Fig. 6d, d’, f). Overall, this suggests that Pdx1 is not required for duct formation, but rather for differentiation of duct-associated, Notch-responsive progenitor cells towards an endocrine cell fate.

### *Pdx1* mutants have disrupted glucose homeostasis

Free glucose in the circulation is maintained within a narrow concentration range to supply immediate metabolic demands, and excess glucose is taken up into cells through the action of Insulin. Since loss of Pdx1 function in mutants reduced beta cell number and blocked new islet cell formation, we next examined how this impacted metabolic homeostasis during further development. We used biochemical measurement of free glucose to indicate the status of glucose regulatory activity, as has been previously described in zebrafish^25^,^44^.

**Figure 4:**
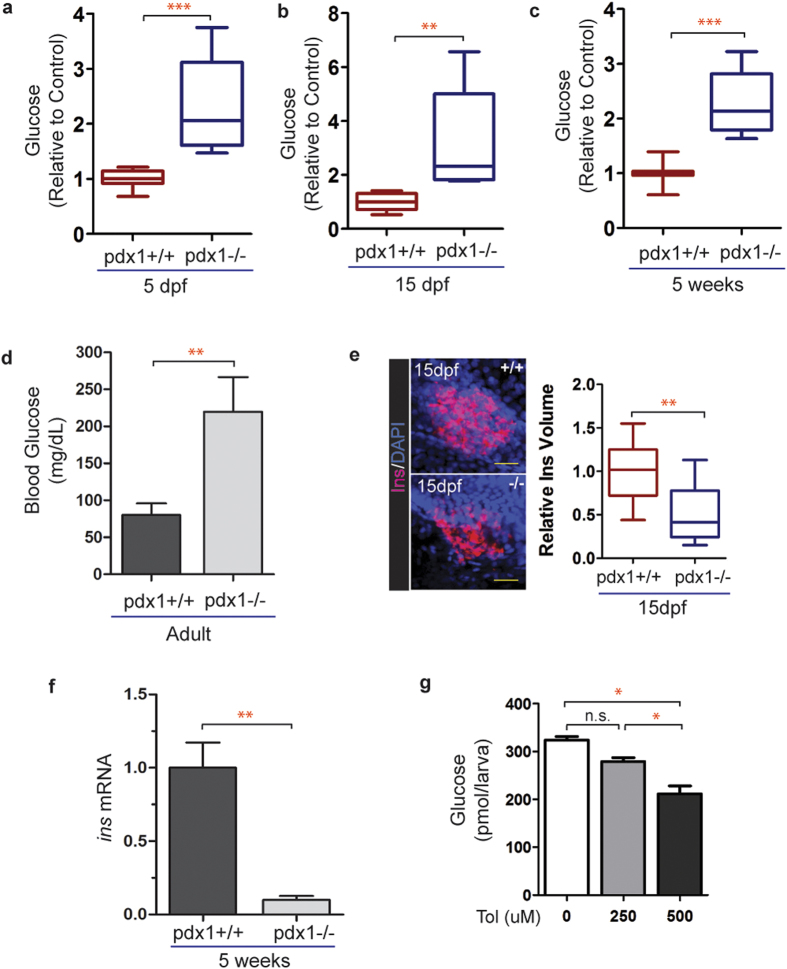
Glucose homeostasis is disrupted in *pdx1* mutants. (**a**) Quantitation of glucose levels from 5 dpf whole larval extracts (pools of 10 embryos). n = 4 biological replicates, results combined from 3 independent experiments (***p < 0.0001, t-test). (**b**) Glucose levels measured at 15 dpf in extracts from pools of 3 fish, normalized to the average control value. n = 4 biological replicates, results are combined from 3 independent experiments (**p < 0.01, t-test). (**c**) Glucose levels relative to control from 5-week single fish (wild type, n = 7; mutant, n = 10, ***p < 0.0001, t-test). In box plots, line shows the median, box extends from the 25^th^ to the 75^th^ percentile, whiskers indicate 10^th^ and 90^th^ percentiles. (**d**) Adult blood glucose levels. Values are mean ± SEM. (*pdx1+/+*, n = 13; *pdx1−/−*, n = 9; **p < 0.01; t-test.) (**e**) Pancreas of wild type and mutant zebrafish at 15 dpf, immunostained with insulin (Ins) antibody, nuclei are counterstained with DAPI (left). Scale bar, 20μm. Quantitation of the volume of insulin staining (right). Results combined from 2 independent experiments. (*pdx1+/+*, n = 15; *pdx1−/−*, n = 16) **p < 0.001 (t-test). (**f**) Relative *insulin* mRNA levels as determined by qPCR in 5-week old wild type and pdx1*−*/*−* mutant fish. **p < 0.01 (t-test) (**g**) Treatment of *pdx1* mutants at 5 dpf with 500 uM tolbutamide for 2 hours lowered glucose levels by 36%. *p < 0.05 (one-way ANOVA, Tukey’s Multiple Comparison Test). Results shown are representative of 2 independent experiments.

We first measured glucose levels at 5 dpf, up to which time nutrients are predominantly provided from the embryonic yolk sac^28^. At 5 dpf, glucose was on average 2.3-fold increased in mutants as compared to controls (Fig. 4a). At 15 dpf, after 10 days of growing in a static water system and receiving a high-nutrient diet (SDS100 fry food and paramecium), glucose levels were 3.2-fold elevated in mutants as compared to controls (Fig. 4b). Elevation of free glucose was sustained in the mutants when measured at 5 weeks of age (2.3 fold, Fig. 4c). Blood glucose levels were 2.7-fold elevated in *pdx1* mutant adults (4–8 months old), averaging 219 mg/dL, as compared to 80 mg/dL in controls (Fig. 4d). Blood glucose measurements obtained from wild type adults were consistent with previously reported values^8^,^45^, while values above 200 mg/dL seen in the *pdx1* mutants were similar to those reported in zebrafish following beta cell ablation^11^,^13^. To examine how glucose dysregulation correlated with insulin expression, we performed insulin antibody staining of the pancreas at 15 dpf. Insulin protein expression was decreased on average by 50% compared to controls (Fig. 4e). At 5 weeks, *insulin* transcript levels in whole animal extracts were reduced by 90% (p < 0.005; Fig. 4f). Overall, elevated glucose levels were seen in *pdx1* mutants from embryonic through adult stages, indicating a persistent hyperglycemic state.

Sulfonylureas are anti-glycemic agents that bind ATP-dependent potassium channels, causing membrane depolarisation, calcium influx and beta cell insulin secretion^46^. They are used to treat T2DM in patients possessing residual beta cell function, and are particularly effective in cases of MODY due to defects in transcription factors important for beta cell development and function^3^. To determine the responsiveness of the hyperglycemia in *pdx1* mutants to pharmacological glucose modulators, we treated 5 dpf mutant larvae with increasing concentrations of the sulfonylurea drug tolbutamide. A 2-hour treatment with 250 uM had minor effects while 500 uM lowered glucose levels by 36% (Fig. 4g). In summary, these data further confirmed similarity of our zebrafish diabetes model to the mammalian system.

### Nutrient effects on the islet in *pdx1* mutants

Overfeeding and obesity contribute to, and exacerbate, the diabetic phenotype in mammals^2^,^47^. Prior studies have shown that overfeeding can induce obesity in adult zebrafish^48^, but similar responses in younger fish have not been demonstrated. Therefore, we analyzed the response of wild type and *pdx1* mutant larvae to overfeeding protocols. Supplementing the standard larval diet of paramecium and larval powder (55% protein, 14% lipid) with increasing concentrations of egg yolk (17% protein, 31% lipid; high fat diet, HFD) from 5 dpf until 11 dpf caused a notable change in larva appearance and a significant increase in body length as compared to larvae fed only larval powder (MIN) diet (Supplementary Fig. 7a, b). Specifically, larva body length increased 15–17% with once daily addition of 5–25 ug/ml egg yolk. We then determined if the HFD similarly affects *pdx1* mutants. With application of our HFD protocol (larval powder, paramecium and 10 ug/ml egg yolk, 1x/day) for 7 days (5dpf to 11dpf), both controls and *pdx1* mutants showed increases in length as well as weight when assessed at 12dpf (Supplementary Fig. 7c, d). The difference in length and weight between MIN versus HFD fed *pdx1* mutant fish was not statistically different from similarly treated controls (Supplementary Fig. 7c, d).

Proliferation of beta cells has been reported in mouse diabetes models as an early response to increased metabolic demand^49^. Cell proliferation in response to nutrient overload was examined by measuring EdU incorporation, compared between larvae left unfed or fed powder plus 20 ug/ml egg yolk solution (HFD) for 48 hours, starting at 5 dpf. Both controls and *pdx1* mutants that were not fed showed rare proliferating beta cells (Supplementary Fig. 8a, left). There was a trend towards increased proliferation with feeding, but this did not reach statistical significance in either group (Supplementary Fig. 8a, right, b).

**Figure 5:**
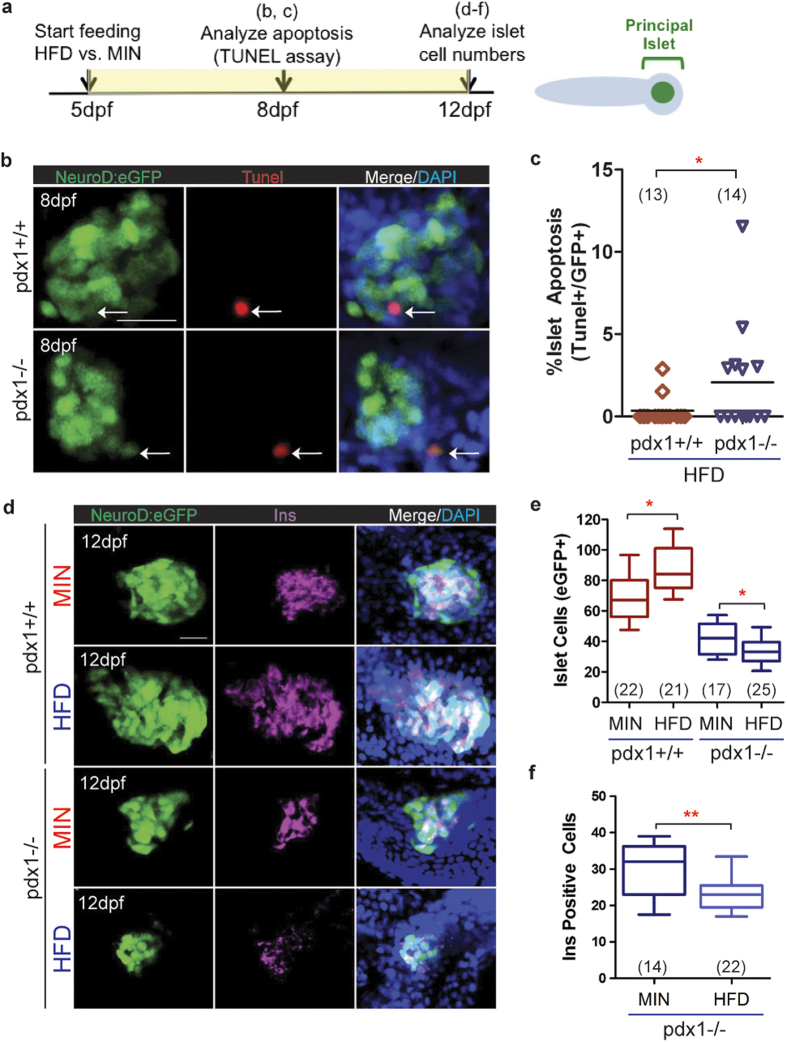
Feeding effects on beta cell induction and viability in *pdx1* mutants. (**a**) Timeline schematic of larval feeding and analyses. (**b**) MIPs of confocal images of the principal islet from *NeuroD:eGFP+* control (top) and *pdx1* mutant (bottom) larvae fed HFD from 5–7 dpf, and analyzed at 8 dpf by TUNEL labeling and immunostaining for GFP. Nuclei were counterstained with DAPI (blue). Arrows indicate eGFP+/TUNEL+ islet cells. Scale bar, 20μm. (**c**) Quantitation of the percentage of apoptotic cells (TUNEL+/GFP+) in the principal islet of wild type and mutant larvae as shown in (**b**). Line indicates median. *p < 0.05, Mann-Whitney, one-tailed t-test. (**d**) Representative MIP confocal images of islets from 12 dpf Neurod:eGFP+ wild type and mutant larvae fed powder (MIN) or high fat diet (HFD) from 5–11 dpf, followed by immunostaining for GFP and Ins (magenta). Nuclei were counterstained with DAPI (blue). Scale bar, 20μm. (**e**) Number of NeuroD:eGFP+ islet cells in 12 dpf larvae treated as in (**d**). For *pdx1+/+* and *pdx1−/−*, comparing MIN to HFD treated, *p < 0.05, Mann-Whitney, two-tailed t-test. (**f**) Number of Ins-expressing cells from mutant larvae as in (**d**) (**p < 0.01; t-test). In box plots, the median is indicated, boxes span the 25^th^ to 75^th^ percentile, whiskers the 10^th^ to 90^th^ percentile. In all graphs, number of larvae analyzed per group (combined from 2 or more independent experiments) as shown.

Later stages of type 2 diabetes show decreased beta cell mass, resulting in large part from increased beta cell apoptosis^47^,^50^,^51^. In larval zebrafish, brief overfeeding with an egg yolk solution activates differentiation of endocrine progenitors, without causing apoptosis^52^. The absence of a toxic effect after nutritional excess was perhaps due to the short duration of the treatment and the vigor of normal beta cells. We therefore examined whether the beta cells of *pdx1* mutants would show altered sensitivity to overfeeding. *NeuroD:eGFP+;pdx1^−/−^* mutant and *NeuroD:eGFP+; pdx1^+/+^* control larvae were fed with powder plus 20 ug/ml egg yolk solution (HFD) for three days (5 dpf to 7 dpf), and islets were examined for apoptotic cells by TUNEL labeling at 8 dpf (Fig. 5a). We quantitated cells co-expressing GFP in the islet, rather than looking for insulin protein, as insulin is often downregulated in beta cells exposed to hyperglycemia^53^,^54^. Furthermore, regions of insulin staining can be difficult to conclusively associate with fragmented cells identified by the TUNEL assay. TUNEL+ cells were identified in the islet in 43% of mutant embryos (n = 14), and less frequently in wild type embryos (15%, n = 13, Fig. 5b). Overall, we detected a rate of 2% apoptotic cells in the islet of HFD-fed mutants as compared to 0.4% in HFD-fed wild type controls (Fig. 5c).

To assess changes in the islet after prolonged overfeeding, *NeuroD:eGFP+;pdx1*^*−/−*^ mutant and *NeuroD:eGFP+;pdx1*^*+/+*^ control larvae were fed a 10 ug/ml egg yolk supplemented HFD for 7 days (from 5 dpf until 11 dpf) or larval powder only (MIN) and examined by immunostaining for GFP+ islet cells and insulin expression (Fig. 5a). Wild type controls fed the HFD had increased eGFP+ positive islet cells and more insulin expression as compared to larvae fed the MIN diet (Fig. 5d,e). By contrast, *pdx1* mutants fed the HFD had fewer eGFP+ cells and reduced insulin expression compared to mutants fed the MIN diet (Fig. 5d,e). The average number of insulin expressing cells was 30 ± 8 cells in MIN fed as compared to 24 ± 6 cells following HFD feeding (Fig. 5f). These observations suggest that the beta cells of *pdx1* mutant larvae are vulnerable to nutrient overload, as manifested by increased islet cell apoptosis and reduced beta cells.

## Discussion

In this work we demonstrate that the *pdx1* mutant zebrafish is a new vertebrate model of diabetes, as fish show the key features of decreased beta cells and insulin, and persistently elevated glucose levels. In agreement with previous studies using morpholino knockdown of *pdx1*^24^,^41^, an initial islet cell population is established in the absence of *pdx1*. Exocrine tissue and duct cells are specified in *pdx1* mutants, but acinar morphology is perturbed and duct-associated progenitors are not competent to differentiate into late endocrine cells. We found elevated glucose at 5 dpf, before external feeding begins, which persisted in larval and juvenile fish, and was maintained in adults. In addition, the high glucose level was reduced by the anti-diabetic drug tolbutamide, and we found evidence for enhanced beta cell sensitivity to nutrient load in the islets of mutants.

Overall, the homozygous phenotype of *pdx1* mutant zebrafish resembles human neonatal diabetes in its early disease manifestation and severe endocrine dysfunction. The finding of endocrine impairment combined with some exocrine perturbation, in contrast to the absence of pancreatic development seen in mammals with a null mutation in *Pdx1*, suggests that zebrafish exocrine tissue is comparatively less susceptible to Pdx1 deficiency. A similar discrepancy in transcription factor dose requirement among vertebrates is seen in comparing zebrafish mutant for *hnf1ba* with human disease caused by mutations in *HNF1B*, which causes MODY5. The hypomorphic *hnf1ba* mutation in its homozygous form in zebrafish causes exocrine hypoplasia and variable disruption of beta cell formation^55^, resembling the human disease which however is caused by heterozygous mutations.

In both mouse and zebrafish, complex spatial and temporal regulation of *pdx1*, in cooperation with *ptf1a, mnx1 (also called hb9* or hlxb9) and other key pancreatic transcription factors, are essential for growth, differentiation and distribution of all pancreatic cell types^56^. Regulatory interactions and dosage effects are conserved but not absolutely identical among vertebrate species. In mouse, the single Mnx1 family member is required early for dorsal bud formation and is critical for beta cell development^56^. In zebrafish, the Mnx1-related gene *mnx2a*, which does not exist in mammals, has a proposed role in exocrine pancreas formation^57^ which could potentially compensate for loss of Pdx1 in some aspects of ventral bud development. Our findings of abnormal exocrine differentation in *pdx1* mutants are consistent with mouse studies demonstrating that Pdx1 expression is required not only for beta cell development but also for complete differentiation of acinar tissue^58^.

A transient early population of insulin- and glucagon-expressing cells is seen in *Pdx1*^*−/−*^ mutant mice^29^, which may be the equivalent of the islet cells in *pdx1* mutant zebrafish. The role of these cells in mammals remains undefined. The glucose elevation in *pdx1* mutants is less pronounced compared to some zebrafish models with more complete beta cell loss, suggesting functional capacity of the early cells. For example, following beta cell ablation in adult zebrafish, glucose levels are elevated up to 5-fold^11^,^13^, which is greater than the approximately 2.5-fold increase seen in *pdx1* mutants. By contrast, a 2.5-fold increase in glucose is seen at 16.5 dpf in an alternative beta-cell ablation transgenic model in which gluose elevation may be dampened by transient insulin production^14^. In these examples, the regenerative response is a confounding factor in the analysis.

Cells with gene expression characteristics of duct-associated Notch-responsive cells (2F11 positive, Nkx6.1 positive) were detected in *pdx1* mutants but they did not form endocrine cells, suggesting that Pdx1 interacts with Notch signaling in the regulation of progenitor differentiation. Further evaluation of this finding will make use of Notch reporter constructs^34^ that can be introduced into the *pdx1* mutant background. In mammals, beta cell proliferation as well as progenitor differentiation can contribute new beta cells during development and under conditions of metabolic stress^47^. We found low rates of beta cell proliferation in unfed larvae, which increased in both wild types and mutants under our high fat feeding conditions, but not to a level of statistical significance. This result implied that mutants engaged a proliferative response to nutrient overload, but this was overwhelmed by loss of cells, as the beta cell number was in fact reduced after one week of HFD feeding. In wild types, the islet increased in size following feeding to an extent that signified contribution from both proliferation and new cell differentiation. A low beta cell proliferation rate is consistent with the report of Maddison and Chen, 2012^52^, although other studies in zebrafish have detected higher levels of beta cell proliferation, and robust stimulation by feeding^14^,^59^. This discrepancy is possibly due to variation in detection method, or to differences in the stage analyzed and feeding regimen applied.

In our study, following an extended time of high nutrient feeding, we detected increased islet cell apoptosis and a reduction of beta cells in *pdx1* mutants during larval stages. This is consistent with the established link between Pdx1 function and beta cell maintenance and survival in mammals^60^,^61^. Transcription factor deficiencies can result not only from inherited mutations, but also from environmental stresses associated with glucotoxicity and reactive oxygen species^62^. Since reduction of PDX1 is commonly seen in T2DM^62^, analyses of beta cell dynamics in the *pdx1*-mutant diabetes model that we describe can provide mechanistic insights that are applicable to more common forms of diabetes.

The reduced body length and weight seen in adult *pdx1* mutants could have several pathophysiological origins. Decreased muscle mass is a recognized feature of diabetes^63^, as insulin promotes protein anabolic pathways. We found that larval mutants increased in size when overfed, and further analyses of diet-invoked responses at the cellular and molecular level may reveal important new insights into protein metabolic pathologies due to insulin deficiency, and may also provide evidence that exocrine dysfunction contributes to the growth deficiency.

To evaluate the activity of insulin-stimulated glucose utilization and storage pathways, we assessed free glucose content in whole animals from larval through late juvenile stages. Due to the lower limits of assay sensitivity, biochemical measurements at earlier stages must be performed on pools of genetically pre-defined offspring. Fish with the *pdx1* mutation survive and breed as homozygotes, enabling evaluation of glucose homeostasis in diabetic embryos as well as larvae and juvenile fish. Previous studies applying diabetic pharmacologics to zebrafish had to do so in combination with agents to elevate glucose levels in order to see significant changes^44^,^64^. By contrast, the sulfonylurea tolbutamide directly lowered glucose levels in *pdx1* mutants. This demonstrates that anti-diabetic agents can be evaluated using *pdx1* mutant zebrafish in a more straightforward approach.

The mutant fish line reported in this study is particularly suited for *in vivo* monitoring of early stages of hyperglycemia-induced pathologies, which can be visualized at their first manifestations and followed over time in the same animal. We demonstrated that metabolic parameters with relevance for diabetes physiology can be assessed in a mutant population that is phenotypically and genetically consistent, during easy to manipulate larval stages. Thus, with *pdx1* mutant zebrafish, pharmacologic effects and toxicities of new diabetes drug candidates can be evaluated at relatively low cost in a vertebrate whole-animal model before moving on to costly and labor-intensive mammalian studies.

## Methods

### Zebrafish breeding and maintanence

Zebrafish (Danio rerio) were maintained and bred according to established protocols^65^. *pdx1*^*sa280*^ heterozygotes from the Zebrafish Mutation Project^27^ were incrossed, then maintained as separate lines (designated *pdx1+/+*; *pdx1+/−, pdx1−/−*). To obtain *pdx1+/−;neuroD:EGFP* fish, *pdx1+/−* heterozygotes were outcrossed to *TgBAC(neurod:EGFP)nl1*^36^ (NeuroD:eGFP) hemizygotes. These were then incrossed to *pdx1−/−* fish to generate *pdx1−/−;NeuroD:eGFP* homozygous mutants. Sibling *pdx1+/+;NeuroD:eGFP* fish were used to generate control embryos. Fish to be harvested at 8 dpf or younger were maintained in egg water (0.3 g/L Coral Pro Salt (Red Sea) in reverse osmosis H_2_O) in petri dishes at 28 °C (20 fish/25 ml) for the duration of the experiment. For studies of diet-induced phenotypes, fish were moved to mouse cages at 5 dpf, at a density of 25 larvae/500 ml egg water. Zebrafish adult and larval standard length was measured as the distance from the snout to the caudal peduncle, as described^66^. Body Mass Index (BMI) was calculated by dividing the body weight (g) by the square of the body length (cm)^48^. Wet mass of larvae was measured in pools of 4–6 larvae placed in pre-weighed 2 mL eppendorf tubes, after removal of water from the tubes. Fish were fasted for 16 hours prior to collection and analysis. This study was approved by the Austrian Bundesministerium für Wissenchaft und Forschung (GZ BMWFW-66.008/0004-WF/II/3b/2014), and all procedures were carried out in accordance with the approved guidelines.

### Special diet feeding and drug treatments

Powdered egg yolk (Backstars, Bellenberg, Germany) was prepared as a 2000x solution in egg water and stored at 4 °C. The solution was vigorously shaken prior to addition to fish cultures to reach final concentrations as indicated. Larvae on the minimal diet (MIN) were fed SDS100 Fry Feed (Scientific Fish Food), consisting of 55% protein and 14% lipid. Tolbutamide was prepared as a 500 mM stock solution in DMSO and treatments were performed in E3 media (5 mM NaCl, 0.17 mM KCl, 0.33 mM CaCl2, 0.33 mM MgSO4).

### Genotyping

Genomic DNA prepared from embryos and adult fin clips was genotyped by PCR followed by restriction digest with DraI (Fermentas), using the following primers:

For CCCCAACGAAGACTACAGCC

Rev ATGGCCTGCAATCAGGAGTTA

The PCR conditions used were 30 cycles of 94 °C for 30 s, 63 °C for 30 s, and 72 °C for 30 s, followed by one cycle of 72 °C for 5 min. The PCR product was digested for 5 hours at 37 °C. The amplified wild type product was 373 bp. A DraI site present in the mutant allele generated a smaller PCR product of 334 bp. Products were separated on a 2% gel.

### Biochemical Assays

Glucose measurements were performed using the Amplex Red Glucose Assay Kit (Invitrogen), according to manufacturer’s instructions. Larval extracts were prepared by sonication in 200 ul of cold PBS. Single 5-week old fish were first homogenized followed by sonication in 500 ul cold PBS. Extracts were spun for 15 min, 12000 rpm at 4 °C to remove debris, and the supernatant was used immediately or stored aliquoted at –80 °C until the assay was performed. Standard curves were generated with every assay. Graphs represent data combined from at least 2 independent experiments. Glucose values were normalized to protein concentration of the extract, and are reported relative to the average control value. To measure blood glucose levels in adult zebrafish, fish were anesthetized and blood collected essentially as described^45^. Blood from the incision was directly applied to a Freestyle Lite glucose test strip, and glucose readings were obtained from the Freestyle Lite glucose meter (Abbott) as per manufacturer’s instructions.

### Histology

Dissected gut from larvae and adults were fixed in 4% PFA in PBS for 2 hours at room temperature, washed in PBS, serially dehydrated in ethanol and embedded in Paraplast (Sigma). 8 μm sections were further processed for immunohistochemistry or stained with hematoxylin and eosin. Immunostained sections were imaged on a Zeiss LSM5 Exciter, H&E stained sections were imaged using a Leica DM5000B.

### Immunohistochemistry

Immunostainings on whole embryos or larvae were performed as previously described^24^ with the modification that for larvae older than 3 dpf, Phospholipase A2 (0.3 ug/ml) was added to ProteinaseK (10 ug/ml) for permeabilization to improve penetration^67^. Sections for immunostaining were incubated for one hour in blocking solution (PBS/1%DMSO/1%BSA/1% Triton), then incubated overnight at 4 °C with primary antibodies diluted in blocking solution. Sections were washed with PBS+ 0.2% Triton. Primary antisera and dilutions were: guinea pig anti-Insulin (1:200, Dako), rabbit anti-Carboxypeptidase A (1:200, Chemicon), rabbit anti-GFP (1:200, Torrey Pines Biolabs), guinea pig anti-Pdx1 (1:200, generous gift from Chris Wright, Vanderbilt University), mouse anti-Nkx6.1 (1:50, DSHB), mouse anti-Glucagon (Sigma) (1:100), mouse anti-2F11 (Abcam), rabbit anti-Somatostatin (1:200, DAKO). Secondary antibodies (1:1000 dilution) were Alexa-conjugated from Invitrogen. Nuclei were labeled by incubation for 30 minutes at room temperature (sections), or overnight at 4 °C (whole embryos or larvae) in 100 ng/ml DAPI. TUNEL labeling was performed as for immunostaining described above, with an additional incubation in TUNEL Detection Reagent (Roche) for 2 hours at 37 °C degrees prior to incubation with the secondary antibody. For labeling of proliferating cells with EdU, the Click-It 488 Kit (Invitrogen) was used. 5 nl of 100 uM EdU (in 5 mM Citric Acid, pH5.0) was injected into the cardinal vein of 5 dpf larvae anesthetized with 0.003% Tricaine. At 7 dpf, embryos were fixed for 1 hour in 4% PFA, permeabilized in 1% DMSO, then the detection reaction was performed as described^33^. Embryos were then incubated in blocking solution and stained with antibodies as described above.

### qPCR

Total RNA was prepared from dissected gut regions of 5 week old fish using Trizol (Ambion), cDNA was prepared using the First Strand cDNA synthesis kit (Thermo Scientific). qPCR was performed in a CFX Connect Real-Time System (BioRad) using the HOT Fire-Pol EvaGreen qPCR Mix Plus (Solis BioDyne). Data shown is the average of 4 biological replicates, presented as gene expression level relative to controls, after normalization to the housekeeping genes *rpl* and *ef1alpha*. PCR primer pair for *insulin* (5′ to 3′): GCCCAACAGGCTTCTTCTACAAC (F), GCAGATTTAGGAGGAAGGAAACCC (R).

### Imaging and image analysis

Larvae for live imaging were anesthetized using 0.003% Tricaine, immobilized in 1.5% low melt agarose and imaged on a Leica DM6000B microscope equipped with a SPOT-RT3 digital camera (Diagnostic Instruments, Inc., Sterling Heights, MI), using a 20X water objective. Fluorescence stack and DIC images were captured using Visiview software (Visitron Systems, Puchheim, Germany). Fluorescence stacks were processed using ImageJ. Stacks were combined to a single image using an extended depth of focus projection^68^. Adjustment of brightness and contrast, applied equally to all images, overexposed the signal of the islet to reveal the weaker signal present in single cells and small clusters located outside of the principal islet. Immunostainings of whole mounts were imaged with a Zeiss LSM5 Exciter confocal laser microscope using a 40X water immersion objective and a z-step ranging from 1–2μm. Stacks of optical sections were combined using a Maximum Intensity Projection (ImageJ). Insulin+, Glucagon+, Hormone+ and NeuroD:eGFP+ cells were quantitated using the PointPicker tool of ImageJ. Antibody signals were examined in combination with DAPI-staining of nuclei to facilitate cell identification. Confocal Images were processed with a median filter to remove speckle noise and assembled into composites using the FigureJ plugin^69^ of ImageJ and Adobe Illustrator. Insulin signal volume was quantitated using Imaris 7.3.0 (Bitplane). In brief, images were processed by smoothing (0.5 um) and local background subtraction, then a contour surface was created that enclosed the fluorescent signal, and the volume in voxels was determined. Signal threshold and object size filtering were applied consistently to all images.

### Statistics

Graphs were generated and statistical analysis was performed using Prism (Graphpad). Significance was tested using the t-test or one-way analysis of variance (ANOVA) and post-test as indicated, with p < 0.05 considered significant. Unless otherwise noted, data presented are representative of at least two independent experiments. Error bars represent standard error (s.e.m.).

## Additional Information

**How to cite this article**: Kimmel, R. A. *et al.* Diabetic *pdx1*-mutant zebrafish show conserved responses to nutrient overload and anti-glycemic treatment. *Sci. Rep.* **5**, 14241; doi: 10.1038/srep14241 (2015).

## Supplementary Material

Supplementary Information
