# Supplementary material for: Diabetic pdx1-mutant zebrafish show conserved responses to nutrient overload and anti-glycemic treatment
Source: Sci Rep. 2015 Sep 18;5:14241. doi: 10.1038/srep14241 (PMC4585597; doi:10.1038/srep14241)
Supplement: Supplementary Information [file srep14241-s1.pdf]

Supplementary Information

Diabetic *pdx1*-mutant zebrafish show conserved responses to nutrient overload and anti-glycemic treatment

Robin A. Kimmel, Stefan Dobler, Nicole Schmitner, Tanja Walsen, Julia Freudenblum, Dirk Meyer

Supplementary figure 1 - Kimmel

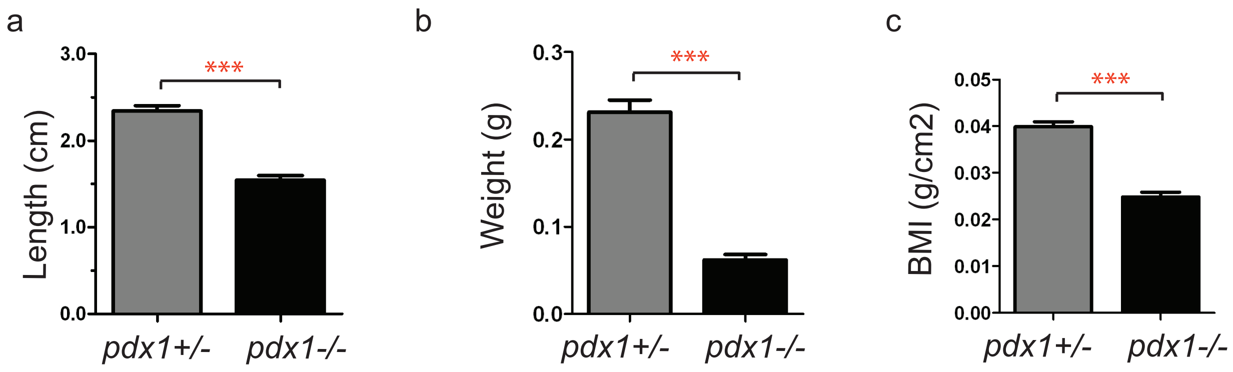

Supplementary figure 2 - Kimmel

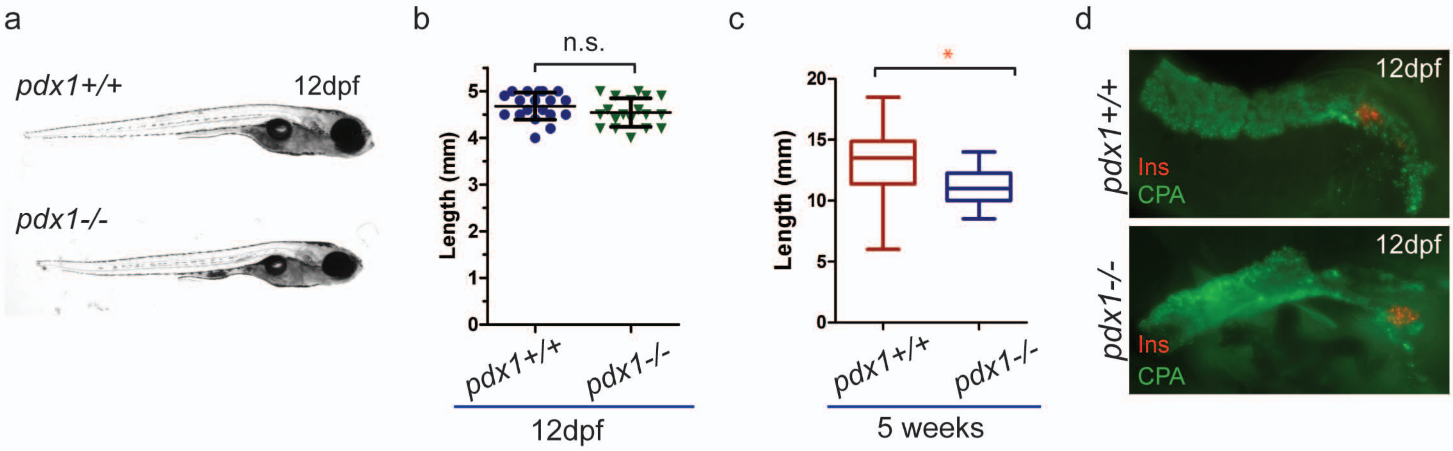

Supplementary figure 3 - Kimmel

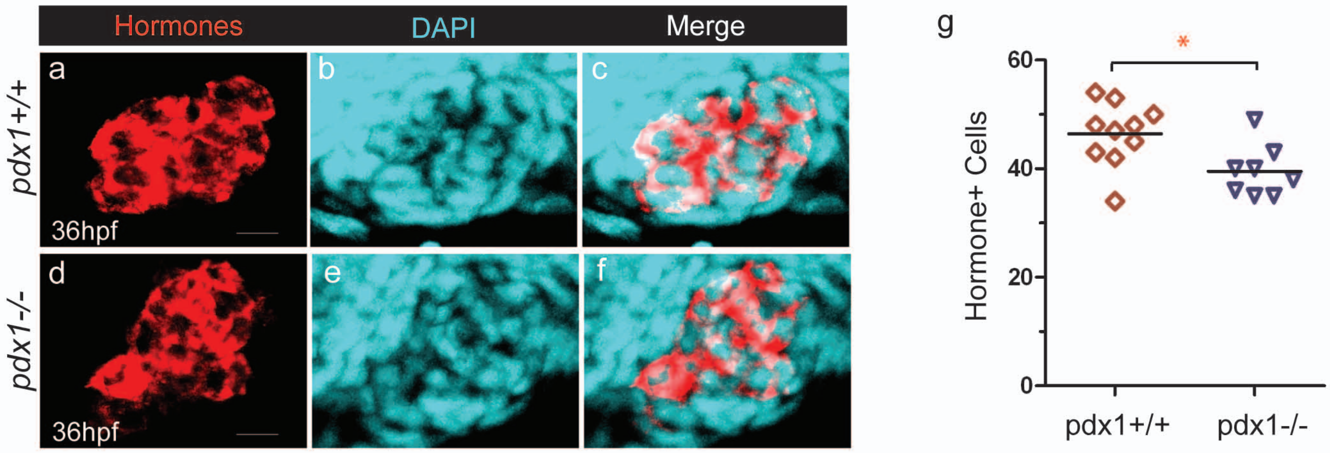

Supplementary figure 4 - Kimmel

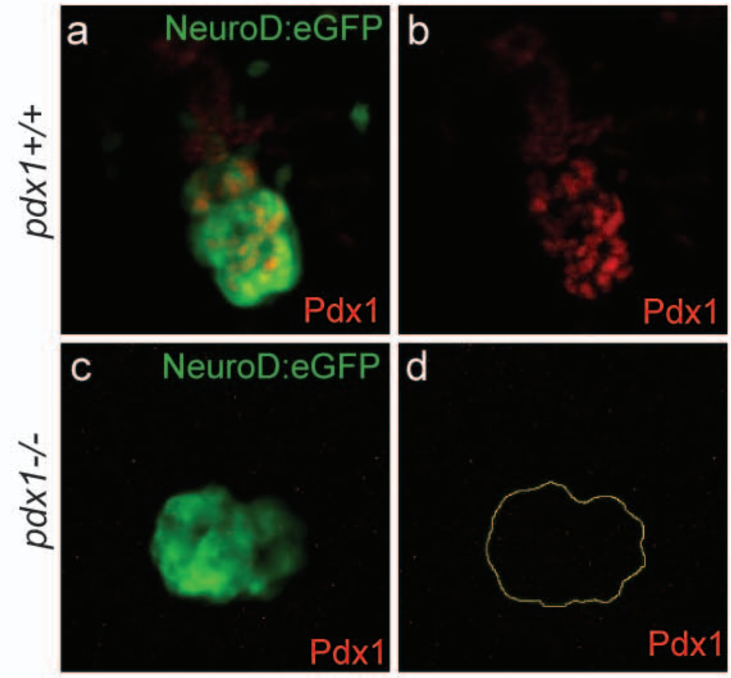

Supplementary figure 5 - Kimmel

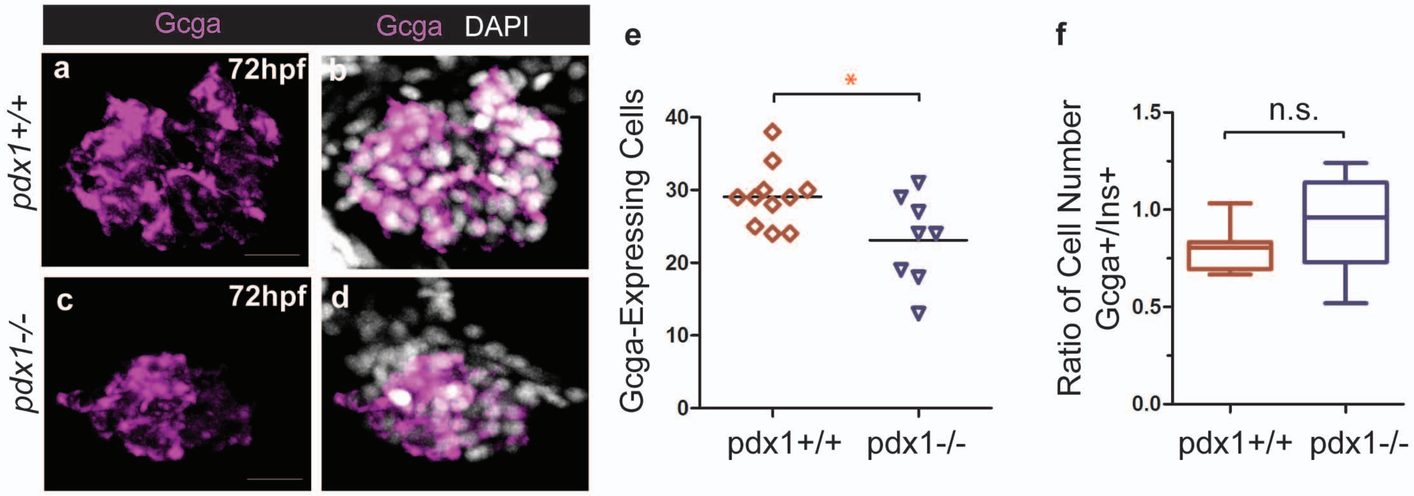

Supplementary figure 6 - Kimmel

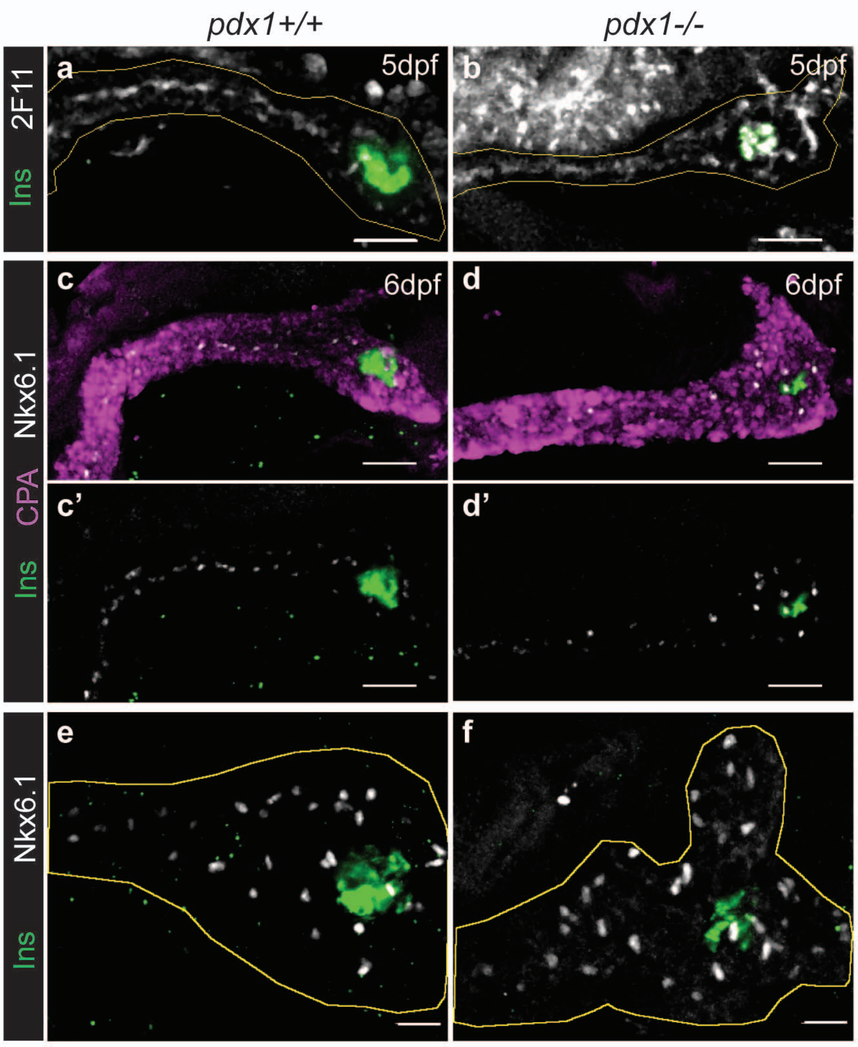

Supplementary figure 7 - Kimmel

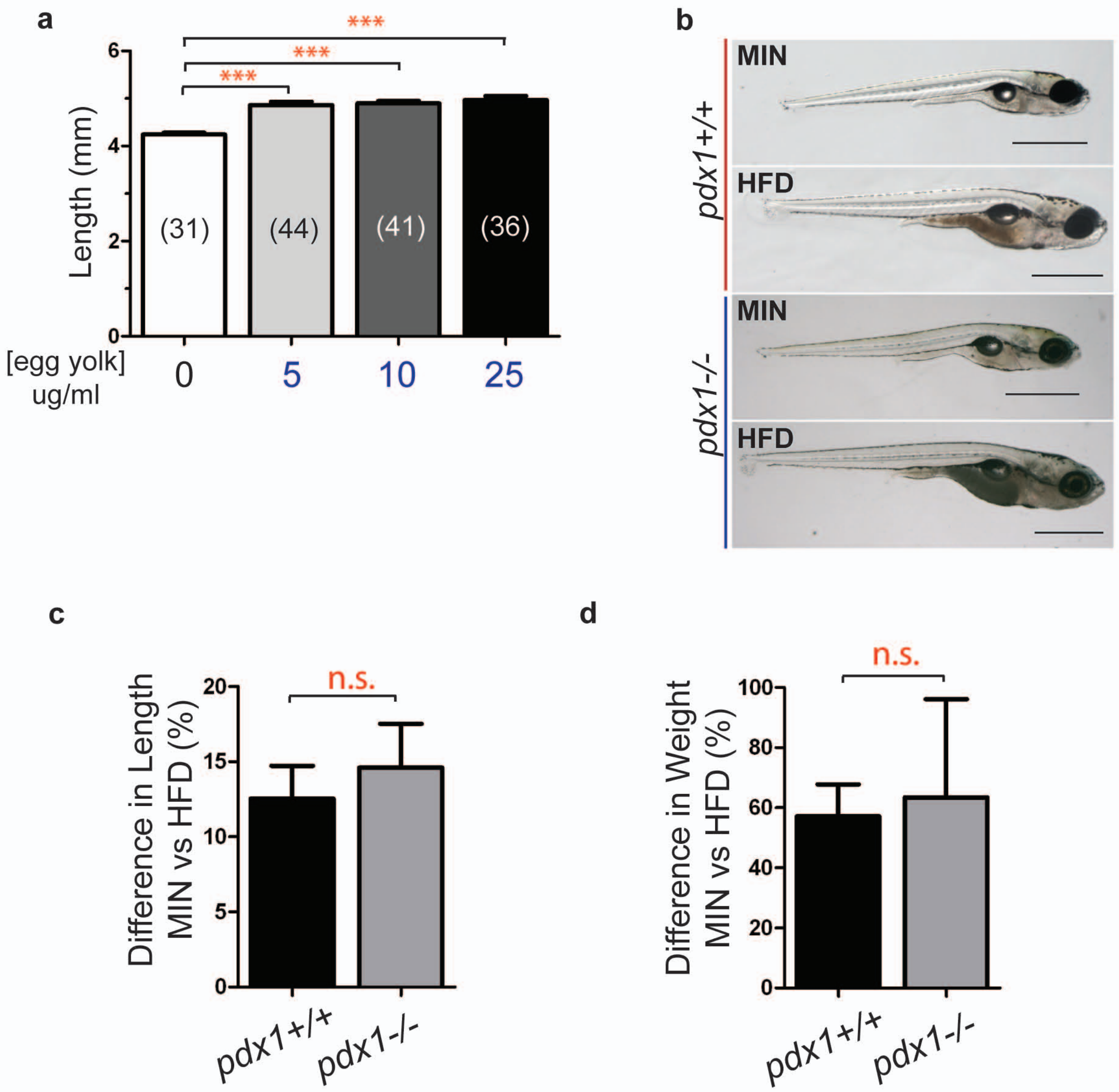

Supplementary figure 8 - Kimmel

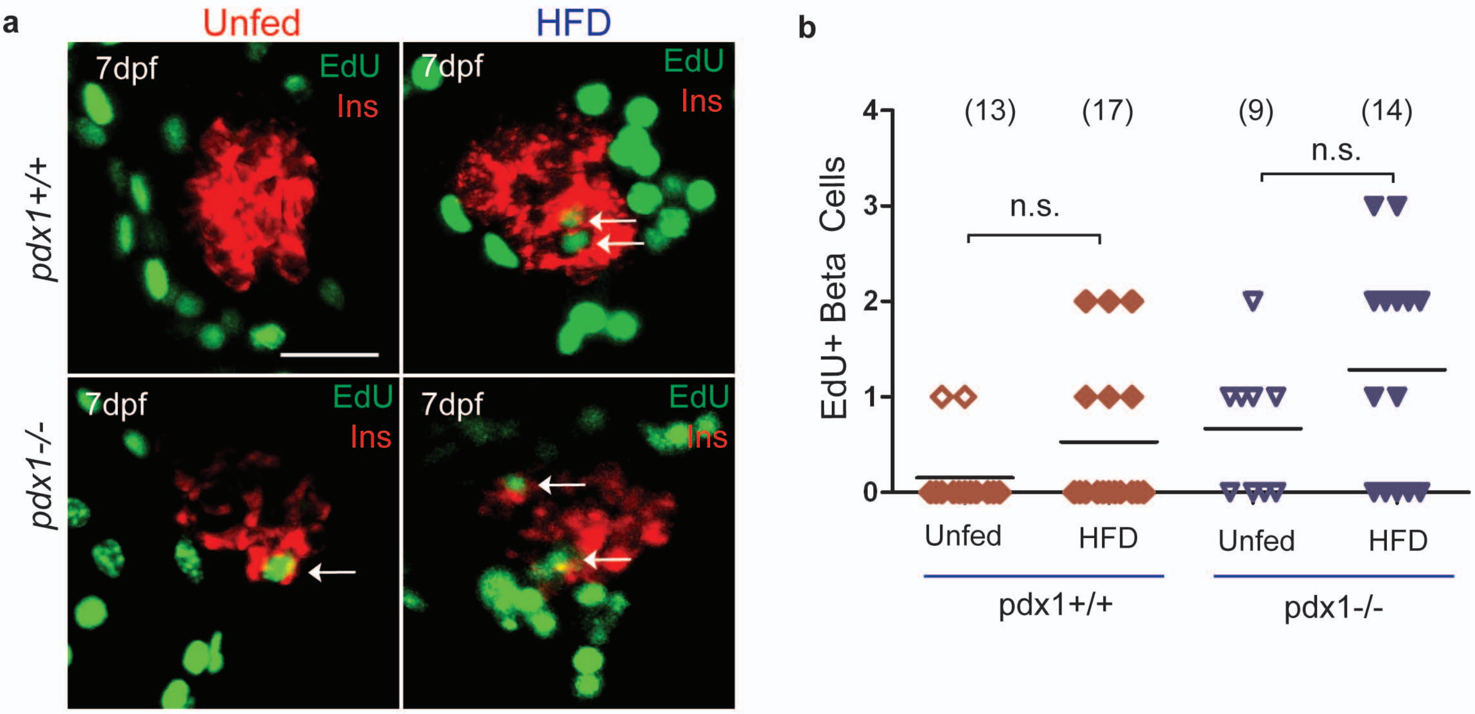

Supplementary Figure Legends

Supplementary figure 1. Reduced size of *pdx1* homozygous mutant adults.

At 3 months of age, *pdx1*$^{-/-}$ mutants (MU) had decreased length (**a**), weight (**b**), and body mass index (BMI, **c**), as compared to *pdx1*$^{+/-}$ heterozygous (HET) siblings from the same clutch. n(*pdx1*+/-) = 35, n(*pdx1*-/-) = 16, ***p<0.0001 (t-test).

Supplementary figure 2. Early growth and development is normal in *pdx1* mutants

(**a**) Brightfield micrographs of representative WT and MU larvae at 12dpf. (**b**) Quantification of standard length at 12dpf. n=19 per group. n.s., not significant (t-test). Error bars show s.e.m. (**c**) Animal length at 5 weeks (*pdx1+/+*, n=12; *pdx1-/-*, n=14; *p<0.05; Mann-Whitney test). The line of the box plots shows the median, the box extends from the 25th to the 75th percentile, whiskers indicate 10th and 90th percentiles.(**d**) Antibody staining of the pancreas at 12dpf to detect exocrine tissue (anti-Carboxypeptidase A, CPA) and beta cells (anti-Insulin, Ins).

Supplementary figure 3. Early endocrine cell formation in *pdx1* mutant embryos.

(**a-f**) Maximum intensity projection of multiple planes from a confocal stacks of the islet at 36hpf in control (**a-c**) and *pdx1* mutant (**d-f**) embryos. Embryos were immunostained with an antibody cocktail against Ins, Gcga and Somatostatin (Hormone+, red) (**a**, **d**) and nuclei were counterstained with DAPI (cyan) (**b,e**). All are dorsal view, anterior is to the left. Scale bar = 10um. (**g**) Quantitation of hormone-expressing cells at 36hpf. n(*pdx1*+/+) = 10, n(*pdx1*-/-) = 8, *p<0.05 (t-test).

Supplementary figure 4. Absence of Pdx1 protein in *pdx1* mutant embryos.

(**a, b**) Pdx1 protein is localized in the islet and extrapancreatic duct in 3.5dpf embryos, as shown by antibody staining in wild type embryos expressing the *NeuroD:eGFP* transgene. Pdx1 protein is not detectable in *NeuroD:eGFP*;*pdx1*$^{-/-}$ mutant embryos (**c,d**). The region

of the islet is outlined in yellow (**d**).

Supplementary Figure 5. Glucagon expression in *pdx1* mutants.

Maximum intensity projections of confocal stacks showing Glucagon (Gcga)-expressing alpha cells (magenta) at 72hpf in control pdx1+/+ (**a**,**b**) and pdx1-/- mutants (**c**,**d**). Nuclei are counterstained with DAPI (gray). Scale bars, 20µm. (**e**) Number of cells expressing Gcga in control (n=11) and mutant (n=8) embryos at 72hpf. Line indicates the mean. *$p<0.05$ (t-test). (**f)** Ratio of alpha to beta cell number (as shown in Figure **3a**). Box plot shows median, boxes span 25th to 75th percentile, whisters 10th to 90th percentile. n.s., the difference in mean value is not significant, t-test.

Supplementary Figure 6. Intrapancreatic duct of *pdx1* mutants.

(**a**, **b**) Projections of confocal stacks of 5dpf larvae immunostained for 2F11 (gray) and Ins (green). (**c**, **d**) Projections of confocal stacks of 6dpf larvae immunostained for Nkx6.1 (gray), Ins (green) and CPA (magenta). (**c'**,**d'**) Same images with CPA channel removed for clarity. Scale bars, 50 µm. Confocal stack projection of head region of the pancreas (outlined in yellow) of 6dpf *pdx1+/+* (**e**) and *pdx1-/-* (**f**) larvae immunostained for Ins (green) and Nkx6.1 (gray). Scale bar, 20µm. In all panels, lateral view with anterior to the right.

Supplementary figure 7. Response of *pdx1* mutants to nutrient excess.

(**a**) Quantification of Standard Length at 12dpf of wild type larvae fed a powder-only (MIN) or egg-yolk supplemented high fat diet (HFD) at the indicated concentrations from 5-11dpf, and analyzed at day 12. n as indicated per group. ***$p<0.0001$ as shown, differences in growth between egg yolk concentrations in the HFD were not significant (Tukey's Multiple Comparison Test). (**b**). Brightfield micrographs of *pdx1* mutant and controls fed a powder-only (MIN) or 10 ug/ml egg-yolk supplemented (HFD) from 5-11dpf, and analyzed at day 12. Scale bar = 1.0mm. (**c**) Relative increase in length for wild type and mutant fish

treated as in (**b**). n.s., not significant (t-test). Results combined from 4 (wild type) and 3 (mutant) independent experiments with 10-25 animals per group. (**d**) Weight (bottom) at 12dpf of fish treated as in (**b**). Fish were weighed in pools of 4-6 animals in 3 (wild type) and 2 (mutant) independent experiments. Results from control and 10ug/ml HFD in (**a**) were included for the calculation in (**c**).

Supplementary figure 8. Proliferative response of beta cells to nutrient excess.

(**a**) Projection of multiple planes through the principal islet from wild type and mutant larvae injected with EdU at 5dpf, not fed or treated with HFD feeding for 48h, followed by analysis for EdU incorporation and immunostaining for Ins at 7dpf. Nuclei were counterstained with DAPI. Arrows indicate EdU+/Ins+ beta cells. Scale bar, 20μm. (**b**) Quantitation of EdU positive beta cells in the islet of wild type and mutant larvae treated as in (**a**). Line indicates median. Number of larvae analyzed per group as indicated (combined from 2 or more independent experiments). Differences between fasting and fed conditions for each genotype are not significant (n.s., Mann-Whitney, one-tailed t-test).
